# Anti-Proliferative Effect of *Allium senescens* L. Extract in Human T-Cell Acute Lymphocytic Leukemia Cells

**DOI:** 10.3390/molecules26010035

**Published:** 2020-12-23

**Authors:** Jiyeon Kim, Dae Han Lee, Bazarragchaa Badamtsetseg, Sangwoo Lee, Soon Ae Kim

**Affiliations:** 1 Department of Medical Laboratory Science, College of Health Science, Dankook University, Cheonan 31116, Korea; yeon@dankook.ac.kr; 2 Department of Pharmacology, School of Medicine, Eulji University, Daejeon 34824, Korea; dleogks120@naver.com; 3 Department of Environment and Forest Resources, Chungnam National University, Daejeon 34134, Korea; batamtsetseg71@gmail.com; 4 International Biological Material Research Center, Korea Research Institute of Bioscience and Biotechnology, Daejeon 34141, Korea; ethnolee@hanmail.net

**Keywords:** *Allium senescens* L., apoptosis, axitinib, dovitinib, leukemia, T-ALL

## Abstract

*Allium* species are well known plants distributed throughout the world, and they contain various bioactive components with different biological activities including anti-cancer effects. In this study, we investigated the inhibitory effect of *Allium senescens* L. (A.S.) extract on cell survival and IL-2-mediated inflammation in human T cell acute lymphocytic leukemia (T-ALL) Jurkat cells. Our results showed that A.S. extract induced caspase-dependent apoptosis of Jurkat cells with no significant cytotoxicity in the normal peripheral blood mononuclear cells. A.S. extract induced ROS generation through the activation of MAPK p38 phosphorylation. It also inhibited IL-2 mRNA expression and NF-κB signaling mediated by phorbol 12-myristate 13-acetate, and phytohemagglutinin. Combined treatment with A.S. extract and axitinib/dovitinib exerted enhanced inhibitory effects on T-ALL cell growth and IL-2 production. These results provide novel information on the potential use of A.S. extract as a therapeutic herbal agent for the treatment and prevention of T-ALL.

## 1. Introduction

Plants containing polyphenolic compounds have been used as sources for the development of medicine. *Allium* L. *genus*, which includes more than 400 species, is distributed around the world, and the plants contain a variety of bioactive compounds such as dietary fibers and polyphenols [1,2,3]. For example, garlic (*Allium sativum* L.) is an aromatic herbaceous plant that is consumed worldwide as food and a traditional remedy for various diseases, and it has been reported to possess several biological properties including anticarcinogenic, antioxidant, antidiabetic, renoprotective, anti-atherosclerotic, antibacterial, antifungal, and antihypertensive activities in traditional medicines [4]. Several studies have reported the potential anticancer effects of *Allium* genus with various mechanisms [4].

*Allium senescens* L. (A.S.) also has been used as a diuretic, antidote, digestive, antiparasitic, and analgesic agent using the stems and leaves in herbal and folk remedies [5]. Though it was reported that A.S. can potentially be developed as therapeutic agents in the treatment of hepatic fibrosis [6], little is known about the bioactivity of A.S. against other diseases such as cancer.

Leukemia is a progressive disease with a poor prognosis and high mortality, characterized by clonal hematopoietic cells in the peripheral blood and bone marrow. Among the hematopoietic diseases, T cell acute lymphoblastic leukemia (T-ALL) is one of the most commonly diagnosed diseases with chronic lymphocytic leukemia (CLL), acute myeloid leukemia (AML), and chronic myeloid leukemia (CML) [7]. T-ALL occurs primarily in children or adults over 40 years of age and is characterized by abnormal white blood cell proliferation in the blood and bone marrow, which interferes with normal immune responses and induces anemia [8]. In particular, interleukin (IL)-2 regulates helper T lymphocyte mediated inflammatory responses in T-ALL [9,10,11]. As a major T-cell growth factor, IL-2 plays a critical role in T cell growth and immune responses mediated by the activation of mitogen activated protein kinase (MAPK) and NF-κB signaling pathways [12,13,14,15]. IL-2 expression is involved in T cell survival and inflammatory responses; hence, the inhibition of IL-2 expression may contribute to the regulation of T cell proliferation and treatment of T-ALL [16,17]. Recently, research on chemotherapy for T-ALL is increasing, but the prognosis is still poor and the problems of side effects persist. In this study, we evaluated the effect of A.S. extract on cell survival and IL-2 expression in human T cell acute lymphocytic leukemia Jurkat cells. The combined treatment effects with tyrosine kinase inhibitors such as axitinib and dovitinib were also observed.

## 2. Results

### 2.1. A.S. Extract Induces Caspase-Dependent Apoptosis of T-ALL Cells

**Figure 1:**
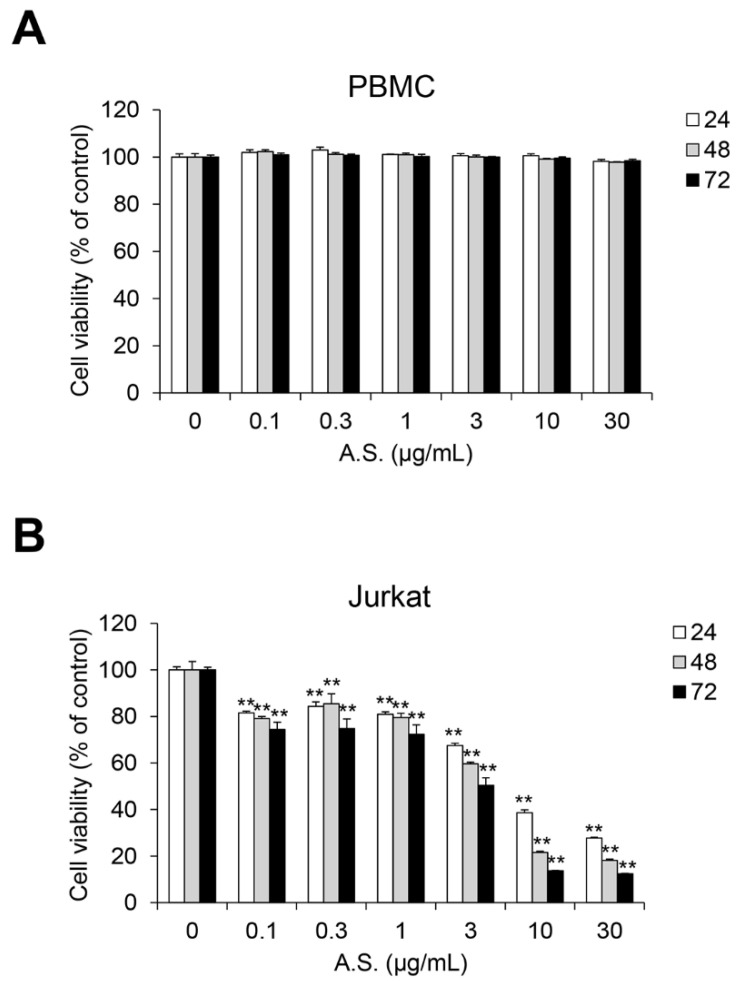
A.S. extract induces cell death in T-ALL Jurkat cells. Peripheral blood mononuclear cells (PBMCs) (**A**) and Jurkat cells (**B**) were incubated with *Allium senescens* L. extract (A.S.) (0–30 μg/mL) for 24–72 h, and cell viability was measured. Experiments were performed in triplicate. Data are presented as mean ± SD. ** *p* < 0.01 (vs. control).

**Figure 2:**
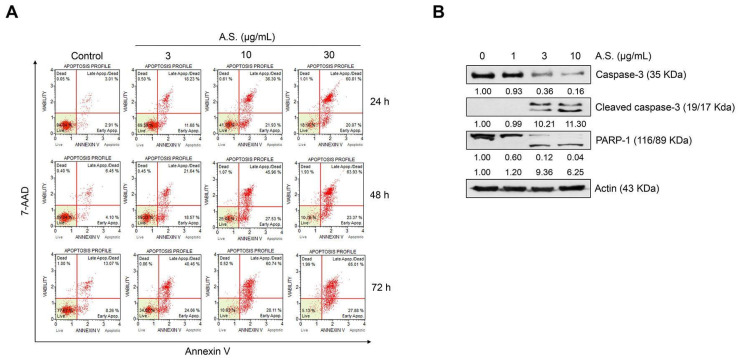
A.S. extract induces caspase-dependent apoptosis in T-ALL Jurkat cells. (**A**) Jurkat cells were treated with A.S. extract for 24–72 h and the apoptotic cells were analyzed using the MUSE® Cell Analyzer. (**B**) After A.S. extract treatment to Jurkat cells for 24 h, protein expression of caspase-3 (cytosol), cleaved caspase-3 (cytosol), and PARP-1 (nucleus) was measured by Western blot using 10% (caspase-3 and actin), 15% (cleaved caspase-3), and 8% (PARP-1) polyacrylamide gels. Actin was used as a loading control against cytosolic and nuclear fractions. The detected band intensities were quantified based on the ImageJ software, and the relative ratio between proteins were normalized by actin.

To determine the optimal concentration of A.S. extract for the cytotoxicity analysis, we conducted cell viability assay in normal PBMCs and T-ALL Jurkat cells. A.S. extract did not show significant cytotoxicity in PBMCs, but Jurkat cell proliferation was inhibited in a dose-dependent manner (Figure 1A,B). Next, we examined the population of A.S.-extract-induced dead cells by flow cytometric analysis and Western blot to determine if A.S. extract induces apoptosis. As shown in Figure 2A, the A.S.-extract-treated group showed a dose-dependent increase in Annexin V- and 7-AAD-positive apoptotic cells. In addition, A.S. extract activated cleavages of caspase-3 and the downstream substrate, PARP-1, in the nucleus (Figure 2B). These results demonstrated that A.S. extract induced caspase-dependent apoptosis in Jurkat cells with no significant cytotoxicity in PBMCs.

### 2.2. A.S. Extract Induces MAPK p38-Mediated ROS Production

**Figure 3:**
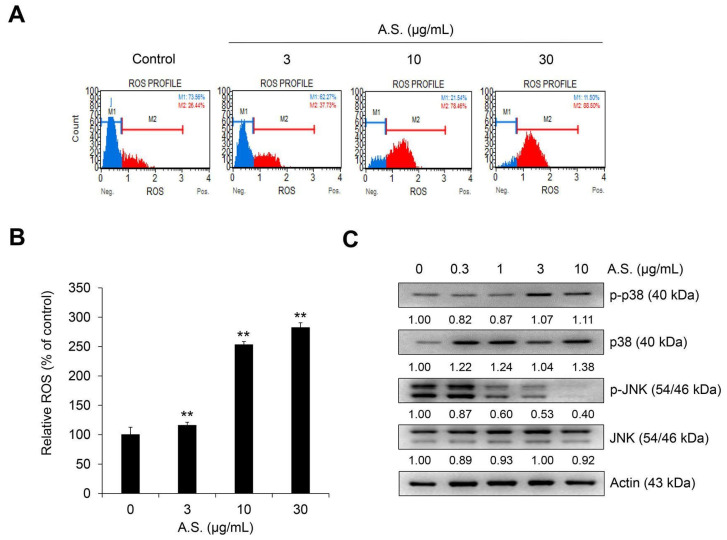
A.S. extract induces reactive oxygen species (ROS) production and the phosphorylation of MAPK p38. Jurkat cells were treated with A.S. extract for 24 h. (**A**) Dihydroethidium stained ROS-positive cells were analyzed using the MUSE® Cell Analyzer and (**B**) the relative percentage of ROS was measured. ** *p* < 0.01 (vs. control). Experiments were performed in triplicate. Data are presented as mean ± SD. Jurkat cells were treated with A.S. extract for 24 h. (**C**) Expressions of p38 and JNK and its phosphorylated forms were measured by Western blot, with actin as a loading control. The detected band intensities were quantified based on ImageJ software (NIH, USA), and the relative ratio between cytosolic proteins was normalized by actin.

As shown in Figure 3A,B, the flow cytometry result shows that A.S. extract dose-dependently increased the population of reactive oxygen species (ROS)-positive cells. Additionally, treatment with A.S. extract activated phosphorylated and total levels of p38, but phosphorylated JNK was suppressed by A.S. extract in a dose-dependent manner (Figure 3C). These results suggest that A.S. extract induced the production of ROS and the activation of MAPK p38 in Jurkat cells. The phosphorylation of p38 or JNK was associated with apoptosis of Jurkat cells [18]. In addition, the balance between p38 and JNK signaling controls apoptosis or autophagy [19]. The inhibition of JNK promotes intracellular ROS production and apoptosis of cancer cells [20]. JNK1 negatively regulates the expression of tumor suppressor p53, but JNK positively regulates the expression of p53 [21]. Although the intracellular mechanism of treatment with small molecule compounds in previous studies and natural products in this study were different, our results suggest that ROS production by A.S. affects p38 activation of Jurkat cells and may be related to cell death by stress-activated protein kinase activity.

### 2.3. A.S. Extract Inhibits the PMA/PHA-Mediated NF-κB Signaling Pathway and IL-2 mRNA Expression and Secretion

**Figure 4:**
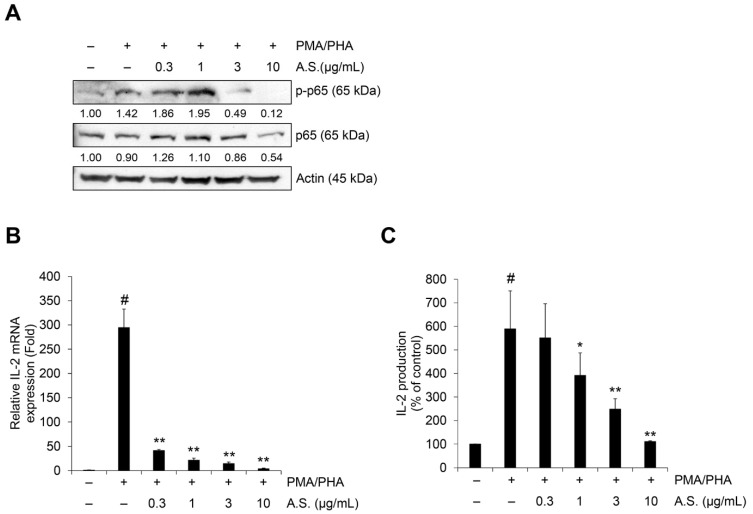
A.S. extract inhibits NF-κB signaling mediated IL-2 expression in T-ALL Jurkat cells. Jurkat cells were treated with A.S. extract phorbol 12-myristate 13-acetate (PMA) (50 ng/mL) plus phytohaemagglutinin (PHA) (1 μg/mL) together for 6 (**A**), 3 (**B**), or 24 h (**C**). (**A**) Expressions of p65 and its phosphorylated form were measured by Western blot, with actin as a loading control. The detected band intensities were quantified based on ImageJ software, and the relative ratio between nucleic proteins was normalized by lamin B1. (**B**) Jurkat cells were pretreated with A.S. extract for 3 h and then treated with PMA (50 ng/mL) plus PHA (1 μg/mL) together for 3 h. Relative IL-2 mRNA expression was measured by qRT-PCR analysis using the 2^−ΔΔCT^ method. (**C**) IL-2 concentration in culture medium of Jurkat cells was treated for 24 h with PMA (50 ng/mL) plus PHA (1 μg/mL) together with A.S. extract. Experiments were performed in triplicate. Data are presented as mean ± SD. * *p* < 0.01 and ** *p* < 0.001 (vs. cells treated with only PMA/PHA); # *p* < 0.01 (vs. untreated cells).

To further evaluate if A.S. extract could inhibit NF-κB signaling and IL-2 expression, its effects on PMA and PHA (PMA/PHA)-induced phosphorylation of p65, a mediator of NF-κB signaling, and IL-2 mRNA expression and secretion were measured. The level of mRNA expression and production of IL-2 was measured based on previous studies (Figures S1 and S2). As shown in Figure 4A, A.S. extract inhibited PMA/PHA-induced nuclear expression of phosphorylated p65 and endogenous p65. In addition, PMA/PHA-induced IL-2 mRNA expression decreased even in the low-dose A.S. treatment (Figure 4B) and PMA/PHA-induced IL-2 secretions in the culture medium were dose-dependently suppressed by A.S. treatment (Figure 4C). These results showed that A.S. extract suppressed the expression and secretion of IL-2 mRNA via the inhibition of the NF-κB signaling pathway in T-ALL Jurkat cells.

### 2.4. Combined Treatment with Tyrosine Kinase Inhibitors Exerts Enhanced Inhibitory Effects on T-ALL Cell Viability and IL-2 Expression

**Figure 5:**
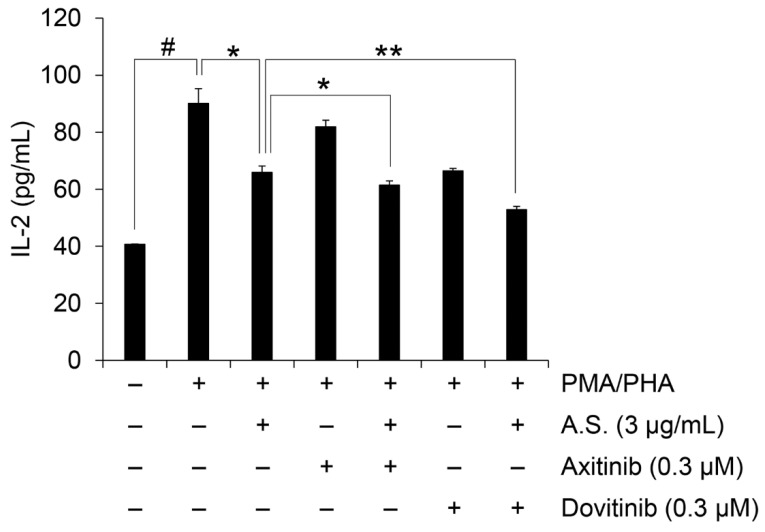
Combination therapy synergistically inhibits IL-2 expression. IL-2 concentration in culture medium of Jurkat cells treated for 24 h with PMA (50 ng/mL) plus PHA (1 μg/mL) together with A.S. extract, A.S. extract alone or in combination with axitinib or dovitinib. Experiments were performed in triplicate. Data are presented as mean ± SD. * *p* < 0.01 and ** *p* < 0.001 (vs. cells treated with PMA/PHA and A.S extract treated group); # *p* < 0.01 (vs. untreated cells).

Cell viability and IL-2 mRNA expression were additionally tested to assess if combined treatment with A.S. extract and tyrosine kinase inhibitors would have synergistic effects in Jurkat cells. To elucidate the synergism of combined treatment, we measured cell viability and calculated combination index (CI) values representing the effects of the combined agents on the cell viability of Jurkat cells, with CI values of one indicating an additive effect and less than one indicating a synergistic effect. As shown in Table 1 and Figure S3, co-treatment with A.S. extract and axitinib or dovitinib showed a synergistic effect on the viability of Jurkat cells. In addition, co-treatment with axitinib or dovitinib showed an enhanced inhibitory effect on the expression of IL-2 compared to the group of A.S. extract only (Figure 5). Overall, these results suggest that A.S. has an inhibitory effect on the expression of IL-2 mRNA through the inhibition of NF-κB signaling. In addition, combination therapy with A.S. extract and conventional tyrosine kinase inhibitors such as axitinib and dovitinib can be used to inhibit T-ALL cell survival and IL-2-relevant immune responses.

## 3. Discussion

For thousands of years, different studies have introduced *Allium species* as potential medicinal agents [19,20,21,22,23,24,25]. There are several reports of multiple biological activities including antioxidant, antibacterial, antiviral, anti-inflammatory, and prevention of cardiovascular disorders from the *Allium* species such as garlic (*A. sativum*) and onion (*A. cepa*) [1,25,26]. Although there are many subtypes and extraction method differences, the biological effects of *Allium* species may be attributable to various compounds including dietary fibers and polyphenolic contents [26,27,28]. For example, it is reported that bulbs of *A. sativum* contain hundreds of phytochemicals including sulfur-containing compounds such as ajoenes (*E*-ajoene, *Z*-ajoene), thiosulfinates (allicin), vinyldithiins (2-vinyl-(4H)-1,3-dithiin, 3-vinyl-(4H)-1,2-dithiin), sulfides (diallyl disulfide (DADS), diallyl trisulfide (DATS), and others that accounted for 82% of the overall garlic sulfur content [4]. Though Shin et al. reported several distinct peaks including *p*-coumaric acid, chlorogenic acid, and rutin as major peaks compared with their respective standards in the HPLC analysis of A.S., the chemical composition and the biological effect of other *Allium* species have not been adequately investigated in different diseases [6].

A previous study demonstrated the anticancer effect of steroidal glycosides isolated from A.S. in human cervical cancer cells [29]. To confirm the anticancer effect in the other types of disorders, we evaluated the effect of A.S. extract on T-ALL cell proliferation and IL-2-mediated immune response. Our study showed that the methanol extract of the leaves and stem of A.S. induced caspase-dependent apoptosis in T-ALL Jurkat cells. This apoptosis inducing effect of A.S. extract might have been mediated by ROS production and the activation of MAPK p38 signaling. ROS are generated through various extracellular or intracellular actions associated with cell growth, differentiation, and cell death [30,31]. The homeostasis of intracellular ROS plays an important role in the determination of cell fate and is affected by several signaling pathways and mechanisms such as NF-κB, mitogen-activated protein kinase (MAPK), and PI3K/Akt signaling [31,32]. In particular, oxidative stress directly or indirectly affects MAPK p38 and JNK phosphorylation through MKK3/6 and MKK4/7 activation, respectively [30,31]. A.S. extract stimulated phosphorylation of p38, but JNK was suppressed. In a cross-talk between ROS and MAPK signaling pathway, internal oxidative stress can suppress the phosphorylation of MKP, subsequently activate JNK, and induce the phosphorylation of MEKK1/2/3/4, MKK3/6, and p38 [30,31]. In addition, it is known that the upregulation of p38 MAPK is involved in T-ALL Jurkat cell death [32]. In this regard, though further experiments will be needed, examining the change in cytotoxic effects and signaling pathway after ROS inhibitors treatment, our results suggest that the production of ROS induced by A.S. extract stimulated the phosphorylation of p38 and inhibited the phosphorylation of JNK. Therefore, the activation of p38 phosphorylation by A.S. extract led to the apoptosis of T-ALL Jurkat cells. In addition, A.S. extract exerted an inhibitory effect on NF-κB signaling and IL-2 expression. IL-2 production by activated T cells promotes T cell proliferation and autocrine signaling, and the regulation of IL-2 expression is important in T-cell-mediated immune system tolerance and auto-immunity control [9]. Additionally, NF-κB signaling is a major mediator in T-cell-mediated immune responses, and it can activate IL-2 production and related inflammatory responses [11,12,13]. Our results showed that A.S. extract may be used for new natural-product-derived drugs development, especially T-ALL.

We also evaluated the anticancer effect of A.S. combined with conventional drugs such as axitinib and dovitinib. Tyrosine kinase inhibitors, axitinib and dovitinib, showed anticancer effects in leukemia cells [33,34,35]. The combined treatment of these tyrosine kinase inhibitors with other chemotherapy exerts effective therapeutic results in leukemia [17,36]. The combination therapy with tyrosine kinase inhibitors such as axitinib and dovitinib may enhance the inhibitory effect of A.S. extract on Jurkat cell proliferation by decreasing PMA/PHA-mediated IL-2 secretion in this study.

In this study, we observed the effect of inhibiting cell growth even at a low concentration, and observed that strong synergistic effects appeared when an anticancer substance such as abitinib were co-treated at a low concentration. Though technical errors in the CCK assay were possible, such a small number of seeding cells (10,000/well), the biological effects of the unknown component of A.S. extracts may be considerable. For example, we observed that low-concentration A.S. treatment decreased IL-2 mRNA expression in PMA/PHA-stimulated T-ALL Jurkat cells.

This study has the following limitations. It is difficult to consider the major substances due to lack of information on the components of A.S. extract, which may explain cytotoxicity and biological effects at low concentration. In addition, we could not determine whether A.S. extract is effective for all T-ALLs, though it effectively exhibited cytotoxicity in Jurkat cells. Though many problems have been experienced by in vitro and in vivo studies, many studies have been conducted on the development of new drugs with natural products and various anticancer drugs have been developed from natural products, including paclitaxel, vinblastine, vincristine, rohitukine, and etoposide [37,38].

## 4. Materials and Methods

### 4.1. Materials

Axitinib and dovitinib were obtained from Selleck Chemicals (Houston, TX, USA). Nuclear and cytoplasmic extraction reagents were purchased from Thermo Fisher Scientific (Waltham, MA, USA). PHA, PMA, and Ficoll Histopaque solutions were purchased from Sigma-Aldrich (St. Louis, MO, USA). Fetal bovine serum (FBS), phosphate-buffered saline (PBS), Dulbecco’s modified Eagle’s medium (DMEM), RPMI 1640 medium, and antibiotics (100 U/mL penicillin and 100 μg/mL streptomycin) were purchased from Corning Life Science (Corning, NY, USA). MUSE^®^ Annexin V and Dead Cell Assay Kit, and Oxidative Stress Assay Kit were purchased from Sigma Aldrich (St. Louis, MO, USA), The Human IL-2 ELISA kit was from KOMABIOTECH (Seoul, Korea), and the Cell Counting Kit-8 was from Dojindo Molecular Technologies (Rockville, MD, USA). Primary antibodies specific for phosphorylated (p)-p65, p65, PARP-1, and actin were purchased from Santa Cruz Biotechnology, Inc. (USA). Antibodies against p-p38, p38, p-JNK, JNK, caspase-3, and cleaved caspase-3 were purchased from Cell Signaling Technology, Inc. (Danvers, MA, USA).

### 4.2. Preparation of Plant Materials

*Allium senescens* L. (*Allium senescens subsp. senescens*) was collected from the Urkhut ovoo, Saikhan soum, Bulgan province, Mongolia. The plant sample was collected and identified by Mr. Zumberelmaa at the Mongolia International University. The voucher specimen was recorded as PB023262, having been deposited at the herbarium of the Korea Research Institute of Bioscience and Biotechnology (Daejeon, Korea). This process was performed in 2008 and methanol extraction was performed in 2014 following the protocol of Bank. Briefly, the freezing dried and refined aerial parts (leaves and stems) of A.S. (91 g) were extracted with 1000 mL of 99.9% (*v*/*v*) methanol with a sonicator (SDN-900H, SD Ultrasonic Cleaner, Seoul, Korea) at 45 °C for 3 days (15 min sonication followed by 2 h standing; repeated 10 times per day). The resultant product was filtered with non-fluorescence cottons, condensed using a rotary evaporator (N1000SWD, EYELA, Tokyo, Japan) under reduced pressure at 45 °C, and lyophilized using a freeze dryer (Christ, Germany). The powder (FBM194–096; yield, 6.36%, approximated as 5.79 g) was dissolved in dimethyl sulfoxide (DMSO) to prepare a stock solution (25.26 mg/mL). The stock solution was diluted with culture medium or DMSO (for in vitro assay) before use in the experiments and DMSO was used as a vehicle.

### 4.3. PBMC Isolation and Cell Culture

The International Review Board of Eulji University (EU 18–13) approved the use of human primary peripheral blood mononuclear cells (PBMCs). PBMCs were isolated from heparinized peripheral blood as previously described [17]. Isolated PBMCs were resuspended in DMEM (10% FBS). Human acute T lymphoblastic leukemia Jurkat clone E6-1 cells were obtained from the Korean Cell Line Bank (Seoul, Korea) and maintained in RPMI 1640 medium (5% FBS).

### 4.4. Cell Viability Assay

PBMCs or Jurkat cells (2.0 × 10^4^ cells/well (0.1 mL)) were seeded into 96-well plates. After 24 h, cells were treated for 24–72 h with A.S. extract alone or combined with axitinib or dovitinib in complete culture media. The Cell Counting Kit-8 was used for measuring cell viability.

### 4.5. Flow Cytometry

Jurkat cells (5 × 10^4^ cells/mL) were treated with A.S. extract (0–30 μg/mL) and incubated for 24–72 h. Annexin V and 7-AAD levels were analyzed with the Dead Cell Assay Kit by the MUSE^®^ Cell Analyzer and MUSE^®^ Annexin V and Dead Cell software module (Merck Millipore). For the quantitative measurements of reactive oxygen species (ROS), the MUSE^®^ Oxidative Stress Kit was also used.

### 4.6. Western Blot Analyses

Total cell lysates were prepared using Nuclear and Cytoplasmic Extraction Reagent (Thermo Fisher Scientific, Waltham, MA, USA). After SDS-PAGE and the transfer to polyvinylidene difluoride (PVDF) membranes, incubation with primary and secondary antibodies for specific protein detection were done and protein was visualized using the Luminata^TM^ Forte Western HRP Substrate (Merck Millipore). Visualized band intensities were measured using Chemidoc™ (Bio-Rad, Hercules, CA, USA). Actin was used as the loading control. Experiments were performed in triplicate. The detected bands were quantified based on the ImageJ software (NIH, Bethesda, MD, USA), and the relative ratio between each sample and loading controls is presented in the figures.

### 4.7. Quantitative Real-Time PCR (qRT-PCR)

Jurkat cells were pretreated for 3 h with A.S. extract alone or combined with axitinib or dovitinib, followed by a 1 h incubation with PMA (50 ng/mL) plus PHA (1 μg/mL) in culture media (5% FBS). Total RNA was isolated using the AccuPrep^®^ RNA Extraction Kit (Bioneer Corp., Daejeon, Korea), and cDNA was synthesized from 100 ng total RNA using the iScript^TM^ cDNA Synthesis Kit (Bio-Rad, Hercules, CA, USA). Quantitative real-time RT-PCR was performed using iQ™ SYBR^®^ Green Supermix (Bio-Rad, Hercules, CA, USA) and the CFX96^TM^ Real-Time PCR System (Bio-Rad, Sacramento, CA, USA). Cycling conditions were as follows: 95 °C for 3 min, followed by 40 cycles of 95 °C for 15 s, 60 °C for 30 s, and 72 °C for 30 s. The following primers were used: IL-2 forward, 5′-ACTTTCACTTAAGACCCAGGGA-3′ and reverse, 5′-AGTGTTGAGATGATGCTTTGACA-3′; GAPDH forward, 5′-GAGTCAACGGATTTGGTCGT-3′ and reverse, 5′-GATCTCGCTCCTGGAAGATG-3′. All reactions were performed in triplicate, and the data were analyzed using the 2^−ΔΔCT^ method [39].

### 4.8. Enzyme-Linked Immunosorbent Assay (ELISA)

Jurkat cells (1.0 × 10^5^ cells/well) were seeded on 24-well plates. After 24 h, the cells were treated with PMA (50 ng/mL) plus PHA (1 μg/mL), and with A.S. extract alone or combined with axitinib or dovitinib in culture media (5% FBS). After an additional 24 h of incubation, the level of IL-2 in the supernatants was measured using human IL-2 ELISA kits following the manufacturer’s instructions.

### 4.9. Analysis of Combined Drug Effects

To determine if the result of the combined treatment was additive or synergistic, combination index (CI) methods, based on the median effect principle of Chou and Talalay, was applied [40,41]. The combined drug effect was analyzed using the CalcuSyn software program (Biosoft, Cambridge, UK).

### 4.10. Statistical Analyses

Data are presented as mean ± SD. Statistical significance was determined using Student’s *t*-test or one-way ANOVA. Differences from controls were considered significant when # *p* <0.01 (vs. the PMA/PHA and A.S. extract untreated cell population); * *p* < 0.01; ** *p* < 0.001 (vs. the cell population treated with only PMA/PHA).

## 5. Conclusions

In this study, we demonstrated the biological activity of A.S. extract on cell proliferation, oxidative stress, and NF-κB signaling-mediated IL-2 expression in T-ALL cells. Although this study was in vitro, the results may provide important information for the development of A.S.-extract-based treatment for leukemia. Furthermore, additional studies including the active compound identification and anticancer effects with animal models will be needed to develop an A.S.-extract-based antileukemic drug.

## Figures and Tables

**Table 1:** Combination index (CI) values for the combination of A.S. and axitinib/dovitinib against Jurkat cells viability.

A.S. (μg/mL)	Axitinib (μM)	Cell Viability (% of Control)	CI Value
0.1	0.1	53.2848	0.0509
0.3	0.3	54.1178	0.1635
1	1	49.6011	0.3768
3	3	40.3136	0.5126
10	10	21.8325	0.2472
30	30	19.3219	0.5259
**A.S. (μg/mL)**	**Dovitinib (μM)**	**Cell viability (% of Control)**	**CI Value**
0.1	0.1	54.7622	0.1108
0.3	0.3	52.7178	0.3747
1	1	38.6599	0.5515
3	3	22.6882	0.5816
10	10	19.1636	0.4195
30	30	19.6665	0.8337

## Data Availability

Data is contained within the article or supplementary material.

## Supplementary Materials

The following are available online, Figure S1: Relative IL-2 mRNA expression after PMA/PHA treatment in Jurkat cells. Figure S2: IL-2 expression after PMA/PHA treatment in Jurkat cells with ELISA. Figure S3: Synergistic cytotoxic effects by co-treatment with A.S. extract and axitinib or dovitinib in Jurkat cells.

## Sample Availability

Samples of the compounds are available from the Korea Research Institute of Bioscience and Biotechnology (Daejeon, Korea).
