# Supplementary material for: Anti-Proliferative Effect of Allium senescens L. Extract in Human T-Cell Acute Lymphocytic Leukemia Cells
Source: Molecules. 2020 Dec 23;26(1):35. doi: 10.3390/molecules26010035 (PMC7795430; doi:10.3390/molecules26010035)
Supplement: Supplementary file 1 [file molecules-26-00035-s001.pdf]

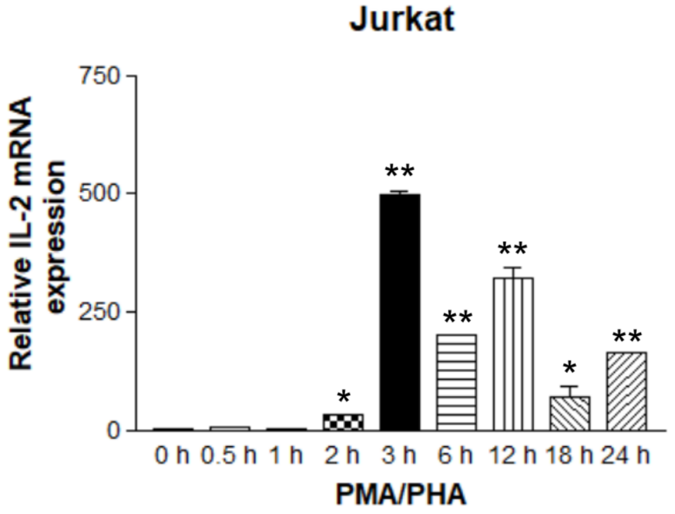

Figure S1. Relative IL-2 mRNA expression in PMA/PHA-treated Jurkat cells. Jurkat cells were treated with PMA (50 ng/mL) plus PHA (1 μg/mL) together for 0-24 h. After incubation, relative mRNA expression of IL-2 was measured by qRT-PCR analysis. Experiments were performed in triplicate. Data are presented as mean ± SD. *$p < 0.01$ and **$p < 0.001$ (vs. untreated control).

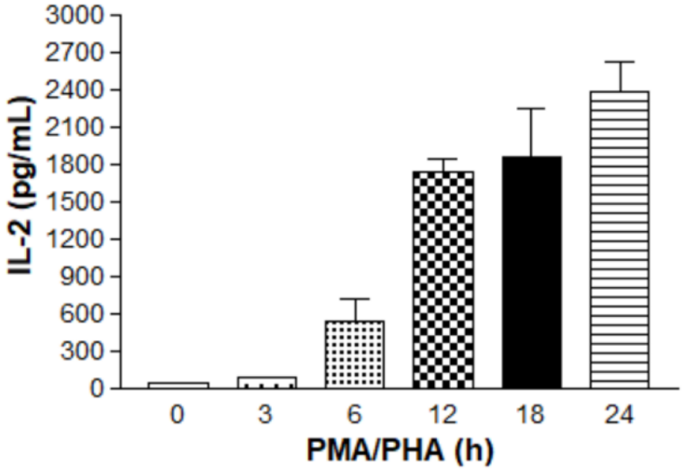

Figure S2. PMA/PHA-induced secretion of IL-2 in Jurkat cells. Jurkat cells were treated with PMA (50 ng/mL) plus PHA (1 μg/mL) together for 0-24 h. IL-2 concentration in culture medium of Jurkat cells were measured by ELISA assay kit. Detailed experimental procedure was described in Materials and Methods section. Experiments were performed in triplicate. Data are presented as mean ± SD.

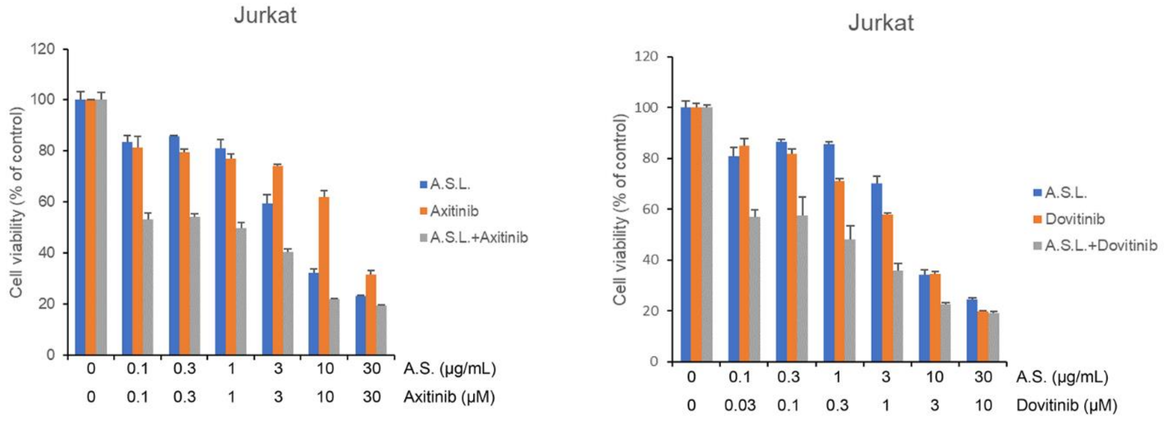

Figure S3. Combination effect of A.S. and axitinib/dovitinib on the cell viability of Jurkat cells. Jurkat cells were treated with A.S. (0–30 μM) and axitinib or dovitinib for 24 h, and then the cell viability was measured by CCK-8 assay. Experiments were performed in triplicate. Data represent mean ± SD.
